# Antioxidant response and related gene expression in aged oat seed

**DOI:** 10.3389/fpls.2015.00158

**Published:** 2015-03-19

**Authors:** Lingqi Kong, Heqiang Huo, Peisheng Mao

**Affiliations:** ^1^Forage Seed Lab, China Agricultural UniversityBeijing, China; ^2^Beijing Key Laboratory of Grassland ScienceBeijing, China; ^3^Department of Plant Sciences, University of California at DavisDavis, California, CA, USA

**Keywords:** oat seed, antioxidant enzymes, proline, storage duration, moisture content, gene expression regulation

## Abstract

To evaluate deterioration of oat seeds during storage, we analyzed oxygen radicals, antioxidant enzyme activity, proline content, and gene transcript levels in oat seeds with different moisture contents (MCs; 4, 16, and 28% w/w) during storage for 0, 6, and 12 months (CK, LT-6, and LT-12 treatments, respectively) at 4°C. The germination percentage decreased significantly with higher seed MCs and longer storage duration. The concentrations of superoxide radical and hydrogen peroxide increased with seed MC increasing. The activities of catalase (CAT), ascorbate peroxidase (APX), and superoxide dismutase (SOD) may have had a complementary or interacting role to scavenge reactive oxygen species. As the storage duration extended, the proline content decreased in seeds with 4 and 16% MC and increased in 28%. These findings suggest that proline played the main role in adaptation to oxidative stress in seeds with higher MC (28%), while antioxidant enzymes played the main role in seeds with lower MCs (4%, 16%). In the gene transcript analyses, *SOD1* transcript levels were not consistent with total SOD activity. The transcript levels of *APX1* and *CAT1* showed similar trends to those of APX and CAT activity. The transcript levels of *P5CS1*, which encodes a proline biosynthetic enzyme, increased with seed MC increasing in CK. Compared with changing of proline content in seeds stored 12 months, *PDH1* transcript levels showed the opposite trend and maintained the lower levels in seeds of 16 and 28% MCs. The transcript level of *P5CS1* was significantly affected by MC, and *PDH1* could improve stress resistance for seed aging and maintain seed vigor during long-term storage.

## Introduction

Deterioration of seeds is a major problem in agricultural production. Seed deterioration depends on the temperature, seed moisture content (MC), and duration of storage (Priestley, 1986; Spanò et al., 2004). The vigor of seeds is reduced or lost during long-term storage, even as stored under low-temperature and low-moisture conditions. The reduction in seed vigor leads to commercial losses and decreased genetic diversity (Lu et al., 2005). Seed aging is associated with certain changes in cellular metabolism and biochemistry, including lipid peroxidation, enzyme inactivation, disruption of membrane integrity, and damage to DNA (McDonald, 1999; Hu et al., 2012). Although the exact mechanisms of seed aging are unknown, the accumulation of reactive oxygen species (ROS), including the superoxide radical ($O_{2}^{\cdot -}$) and hydrogen peroxide (H_2_O_2_), has been suggested to be the major cause of seed deterioration (Lehner et al., 2008; Yao et al., 2012). It has been hypothesized that seeds germinate completely only when the ROS content is maintained below a critical threshold (Bailly et al., 2008). Malondialdehyde (MDA) is a product of ROS, and is a biomarker of oxidative damage (Bailly et al., 1996). To minimize the damaging effects of ROS, antioxidant enzymes such as superoxide dismutase (SOD), catalase (CAT), and ascorbate peroxidase (APX) scavenge ROS (Moller et al., 2007). The amount of ROS is linked to the rate of their production and the capacity of the antioxidant system (Esfandiari et al., 2007). Overexpression of genes encoding SOD, APX, and CAT in tobacco, wheat, and *Arabidopsis* resulted in enhanced seed longevity (Melchiorre et al., 2009; Li and Yi, 2012). Proline has also been shown to scavenge ROS (Smirnoff and Cumbes, 1989). In many plant species, proline accumulation is one of the main metabolic responses to abiotic stress (Evers et al., 2010; Irina et al., 2012). Early studies on proline established a model whereby stress caused the transcriptional up-regulation of the gene encoding Δ-1-pyrroline-5-carboxylate synthetase1 (*P5CS1*), which catalyzes the first two steps of proline biosynthesis (Armengaud et al., 2004), and down-regulation of the gene encoding proline dehydrogenase (*PDH1*), which catalyzes the first step of proline catabolism (Kiyosue et al., 1996; Miller et al., 2005). Both genes were necessary and sufficient for stress-induced proline accumulation.

Oat (*Avena sativa* L.) is the fifth largest cereal crop in the world, with an annual yield of approximately 700 000 tons. Compared with other cereals, oat seed has higher concentrations of soluble fiber, vitamins, minerals, antioxidants, and high quality protein. Therefore, it is an important cereal in terms of its nutritional value (Klose and Arendt, 2012). In recent years, oat has been cultivated more widely, and has become an important forage grass in alpine regions where other grasses cannot grow. The lager deterioration occurs in oat seeds with 10 years storage (Magdalena et al., 1999). The oat seed deterioration results in greater losses in seed vigor, causing great economic losses (Price, 1975; Heneen et al., 2008). The oat seeds have higher lipid content than other cereals such as wheat, maize, rice, and barley. This high lipid content results in faster deterioration of oat seeds (Pekka et al., 2003). The seed MC is another key factor affecting seed vigor during storage. The germinability lost in untreated oat seed was found to depend on its temperature and water content at storage condition (Machacer et al., 1961; Kong et al., 2014). However, the physiological and transcriptional changes in oat seeds with different MCs during storage are unknown.

In this study, we examined the dynamics of ROS, antioxidant enzyme activity, proline content, and gene transcript levels of *APX1*,* CAT1*,* SOD1*,* P5CS1*, and* PDH1* in deteriorated oat seeds with different MCs during storage. The aims were to investigate whether storage treatments at low temperature (4°C) affected ROS levels via its effects on the enzymatic system and proline content, and to evaluate the transcript level of genes encoding antioxidant enzymes during seed aging.

## Materials and Methods

### Plant Material

Oat seeds (Lot#P708O2498) were purchased from the Lockwood Seed and Grain Company (Woodland, CA, USA). The experiments were initiated in May, 2012. The seeds were initially at 8.8% MC and germinated 98% (ISTA, 2012).

### Determination of Seed MC

Seed MC was measured according to the ISTA Procedure (2012). Briefly, approximately 4.5 g seeds were placed in a sample container, weighed, dried at 130–133°C for 1 h, and then reweighed. Four replicates were evaluated for each sample.

### Adjustment of Seed MC

To adjust the MC of the oat seeds to 4, 16, and 28% (w/w) before storage, seeds (~25 g) with 8.8% MC were placed in an aluminum foil bag and then subjected to rehydration or dehydration to achieve the desired water content. The seed MCs were adjusted to 16 and 28% by adding appropriate amounts of distilled water into the foil bags and incubating the seeds at 5°C for 48 h. The seeds were adjusted to 4% water content by desiccation.

### Storage and Germination Assay

Seeds with different MCs were stored at 4°C for 0 months (CK), 6 months (LT-6), and 12 months (LT-12). The seed germination percentage was determined by standard germination tests according to the ISTA protocol (ISTA, 2012). Four replicates of 100 seeds each were germinated in 150-mm Petri dishes on filter paper hydrated with 14 ml water. Germination tests were conducted in a growth chamber (Bio Chamber-Enconair, Winnipeg, MB, Canada) at 20°C under an 8-h light/16-h dark photoperiod. The number of normal seedlings was recorded after 10 days. The seed germination percentage was expressed as the percentage of normal seedlings determined as described in the ISTA protocol (ISTA, 2012).

### Determination of Superoxide Anion ($O_{2}^{\cdot -}$) Production Rate

The $O_{2}^{\cdot -}$ production rate was measured as described elsewhere (Elstner and Heupel, 1976). Seed embryos (1 g) were ground in liquid nitrogen, homogenized in 7 ml phosphate buffer (50 mM, pH 7.8), and then centrifuged at 16 000 rpm for 10 min. The supernatant was centrifuged again. Then, 1 ml supernatant was mixed with 0.9 ml phosphate buffer (50 mM) and 0.1 ml hydroxylamine hydrochloride. The mixture was incubated at 25°C for 30 min, and then mixed with 1 ml sulfanilic acid (17 mM) and 1 ml α-naphthylamine (7 mM); the mixture was incubated at 25°C for 20 min and then the absorbance at 530 nm was recorded.

### Determination of Hydrogen Peroxide (H_2_O_2_) Content

To measure the H_2_O_2_ content, embryos (200 mg) were ground in liquid nitrogen, homogenized in 2.0 ml cold acetone, and then centrifuged at 16 000 rpm for 10 min. The supernatant (1.0 ml) was mixed with 100 μl 10% (w/v) titanium tetrachloride and 200 μl ammonia water, and then mixed well. The mixture was centrifuged at 3000 rpm for 10 min, and the supernatant was discarded. The pellet was dissolved in concentrated sulfuric acid, and then absorbance at 415 nm was recorded. A standard curve was prepared by diluting a 100 μmol/L H_2_O_2_ stock solution to 10, 20, 40, 60, 80, 100 μmol/L.

### Determination of Antioxidant Enzyme Activities

To extract enzymes, embryos (200 mg) were ground in liquid nitrogen, homogenized in 2 ml phosphate buffer (50 mM, pH 7.0), and then centrifuged at 15 000 rpm for 20 min at 4°C. The supernatant was used in the antioxidant enzyme assays.

The activity of SOD was determined according to the method of (Beaucham and Fridovic, 1971). The 3 ml reaction mixture contained 13 mM methionine, 1.3 μM riboflavin, 63 μM nitroblue tetrazolium (NBT) in 50 mM phosphate buffer (pH 7.8), and 25 μl enzyme extract. The enzyme extract was replaced with phosphate buffer in two controls. The reaction mixtures were incubated in a growth chamber (LRH-250-GII, Ningbo, China) at 25°C under illumination. Identical tubes that were not illuminated served as blanks. After illumination for 17 min, absorbance was measured at 560 nm.

The activity of APX was measured in the presence of 0.25 mM ascorbic acid and 10 mM H_2_O_2_ by monitoring the decrease in absorption at 290 nm. The reaction mixture consisted of 200 μl supernatant, 3.4 ml phosphate buffer, 200 μl ascorbic acid, and 200 μl H_2_O_2_. Enzyme activity was determined by measuring the change in absorbance at 290 nm every minute.

To measure CAT activity, 50 μl supernatant was mixed with 3.4 ml phosphate buffer (25 mM, pH 7.0, containing 0.1 mM EDTA), and 200 μl H_2_O_2_. Enzyme activity was determined by measuring the change in absorbance at 240 nm after 1 min.

### Determination of Proline Content

To measure proline content, embryos (200 mg) were ground in liquid nitrogen, homogenized in 2 ml 3% sulfosalicylic acid, and then centrifuged at 4 000 rpm for 5 min. The supernatant (200 μl) was mixed with 200 μl glacial acetic acid and 200 μl acidic ninhydrin, and then mixed well. The mixture was incubated at 100°C for 60 min. The reaction was terminated by placing the mixture on ice, and then extracting the sample with 1.0 ml toluene. The absorbance of the mixture was measured at 520 nm. To prepare the standard curve, an l-proline stock solution (100 μg/ml) was diluted to 1, 3, 5, 7, 9, 11 μg/ml.

### Gene Transcript Analyses

Total RNA was isolated from seed embryos using the phenol–chloroform method (Cooley et al., 1999). Briefly, seed embryos (100 mg) were ground in liquid nitrogen and homogenized in 2 ml RNA extraction buffer (15 ml TLE buffer, 15 ml phenol, 3 ml chloroform) with 30 μl β-mercaptoethanol. The mixture was shaken for 30 min at 300 rpm at 4°C, and then centrifuged at 10 000 rpm for 2 min. The supernatant was collected and mixed with an equal volume of phenol–chloroform–isoamylalcohol (25:24:1), then shaken for 10 min at 4°C and centrifuged for 2 min at 10 000 rpm at 4°C. The supernatant was collected and mixed with an equal volume of chloroform–isoamyl alcohol (24:1), then shaken for 10 min at 4°C and centrifuged for 5 min at 10 000 rpm at 4°C. The supernatant was collected and mixed with a one-third volume of 8 M LiCl, mixed thoroughly, and then incubated at –20°C overnight. The next day, the sample was thawed and then centrifuged at 10 000 rpm for 3 min. The pellet was washed with 1.0 ml 80% (v/v) ethanol. The RNA was dried completely, and then dissolved in 50 μl diethyl pyrocarbonate water. The RNA quality was assessed by 1% agarose gel electrophoresis.

Candidate and reference gene sequences corresponding to the top BLAST hits were identified at the Compositae Genome Project EST database based on sequence homology to known candidates in *Arabidopsis*, and from existing oat sequence data in GenBank. Primer sequences for qRT-PCR analyses were designed using Primer Express (Applied Biosystems, Foster City, CA, USA). The genes and primers used for qRT-PCR analyses are shown in **Table 1**.

**Table 1:** Primers used in real-time PCR.

Accession no.	Gene name	Gene description	Forward primers	Reverse primers
AT2G39800.1	P5CS1	Biosynthesis of proline	TGTCCTCTGGGTGTTCTCTTGAT	CGAATGGCTAAAGACGCAATC
AT3G30775	PDH1	Proline oxidase	CCCCGTGGAGCACATCAT	AAGGTTGAAGCAGAGAGCAATCC
AT1G20630	CAT1	Catalase 1	CAGGCTGGCGAGAGATTCC	AGCATCCGTGAGTGCATCAA
AT1G07890	APX1	Ascorbate peroxidase 1 (APX1)	GCTCCGTGAAGTAAGTGTTATCAAAC	CCTGGGAAGGTGCCACAA
AT1G12520	SOD1	Copper–Zinc Superoxide dismutase 1 (SOD1)	CACAAGCACTTCACAGGAACAGT	TGCCACTCTGAACATTTCATCAC
AT2G37620	ACTIN1	Actin	GCTATTCAAGCCGTGCTTTC	AGCATGTGGAAGGGCATAAC

Total RNA was reverse-transcribed and DNase-treated using a QuantiTect Reverse Transcription Kit (Qiagen, Hilden, Germany). The cDNAs of housekeeping genes and genes of interest were PCR-amplified with an Applied Biosystems 7300 Real-Time PCR System using SYBR Green detection. For each sample, the change in fluorescence was analyzed using DART PCR 1.0 software.

### Statistical Analyses

Data were subjected to analysis of variance (ANOVA) using SPSS for Windows ver. 13.0. Duncan’s multiple range tests (*P* = 0.05) were used to compare treatment means of germination and physiological indicators.

## Results

### Changes in Germination Percentage in Oat Seeds during Storage

**FIGURE 1:**
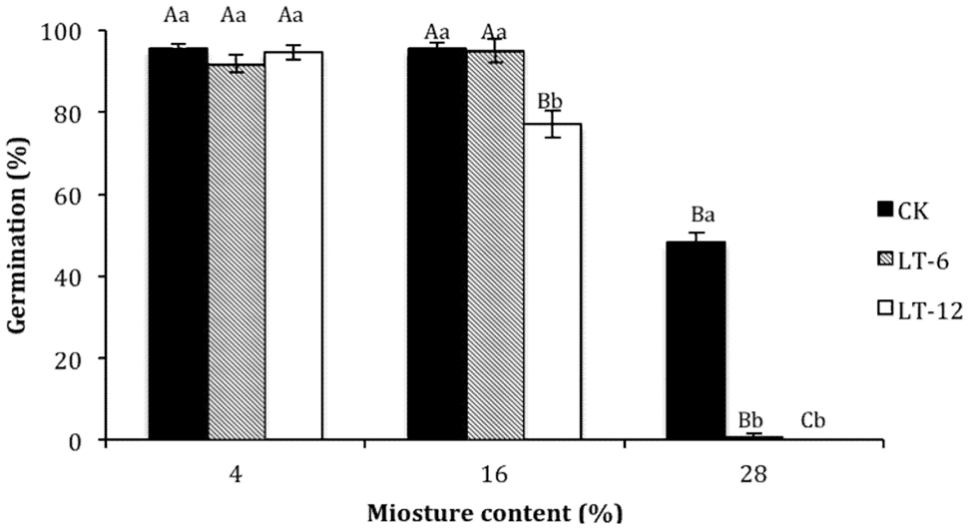
**Effect of storage duration on germination percentage of oat seed with different moisture content (MC) at 4°C**. The different letters indicate significant differences at 0.05 level among treatments as determined by the Duncan’s multiple range test. Means with different capital letters indicate the significant differences of seed at different MC and same storage duration, and with different lowercase letters at the same MC and different storage duration. Data represent the mean ± SD of four or more replicates.

The germination percentage of oat seeds decreased with seed MCs increasing (**Figure 1**). In the storage treatments of CK and LT-6, the germination percentage decreased significantly (*P* < 0.05) at 28% MC. In LT-12, the germination percentage decreased significantly (*P*< 0.05) with MC increasing from 4 to 28%.

The germination percentage of oat seeds with 4% MC did not change significantly (*P* > 0.05) during storage (**Figure 1**). At 16% MC, the germination percentage of seeds in LT-12 decreased significantly (*P* < 0.05). The germination percentage of seeds in LT-6 and LT-12 was zero at 28% MC (**Figure 1**).

### Changes in Superoxide Anion ($O_{2}^{\cdot -}$) Production Rate in Oat Seeds during Storage

**FIGURE 2:**
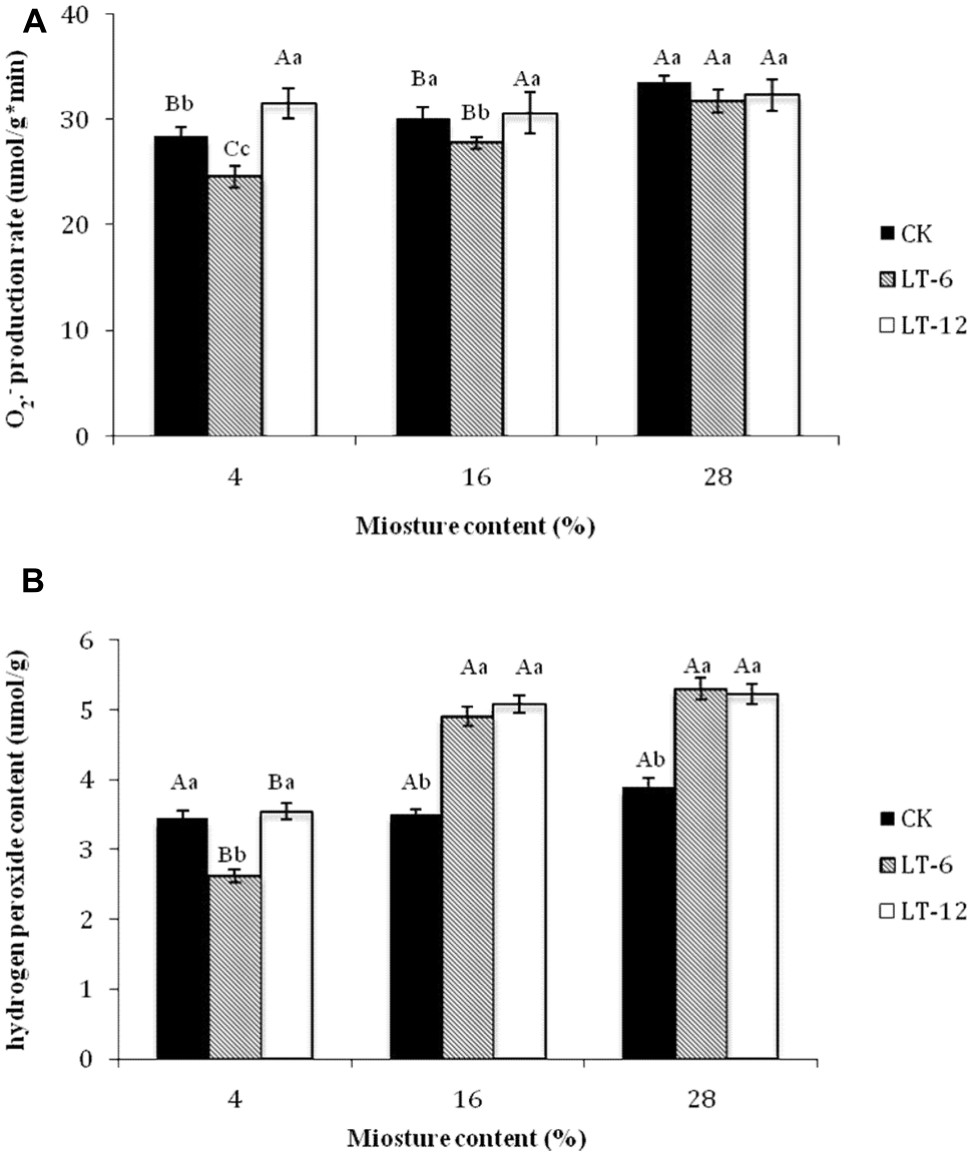
**Effect of storage duration on $O_{2}^{\cdot -}$ production rate and H_2_O_2_ content of oat seed with different MC at 4°C. (A)** $O_{2}^{\cdot -}$ production rate; **(B)** H_2_O_2_ content. The different letters indicate significant differences at 0.05 level among treatments as determined by the Duncan’s multiple range test. Means with different capital letters indicate the significant differences of seed at different MC and same storage duration, and with different lowercase letters at the same MC and different storage duration. Data represent the mean ± SD of four or more replicates.

In the storage treatments of CK, the $O_{2}^{\cdot -}$ production rate increased significantly (*P* < 0.05) at 28% MC. The $O_{2}^{\cdot -}$ production rate in the LT-6 treatment increased significantly (*P* < 0.05) with MC increasing, but not in the LT-12 treatment (**Figure 2A**).

In the seeds with 4 and 16% MC, the $O_{2}^{\cdot -}$ production rate decreased and then increased during storage; the lowest rate was after the stored 6 months (**Figure 2A**). For seeds with 28% MC, there were no significant (*P* > 0.05) differences for $O_{2}^{\cdot -}$ production rate among CK, LT-6, and LT-12 treatments.

### Changes in Hydrogen Peroxide (H_2_O_2_) Contents in Oat Seeds during Storage

In the storage treatments of CK, there was not significantly (*P* > 0.05) different for H_2_O_2_ content of oat seeds among different MCs (**Figure 2B**). In the LT-6 and LT-12 treatments, the H_2_O_2_ contents of seeds with 16 and 28% MC were significantly higher (*P* < 0.05) than those with 4% MC. For seeds with 4% MC, the lowest of H_2_O_2_ content presented after stored 6 months. For 16 and 28% MCs, the H_2_O_2_ content in LT-6 and LT-12 were significantly higher (*P* < 0.05) than those in CK (**Figure 2B**).

### Changes in Activities of Antioxidant Enzymes in Oat Seeds during Storage

**FIGURE 3:**
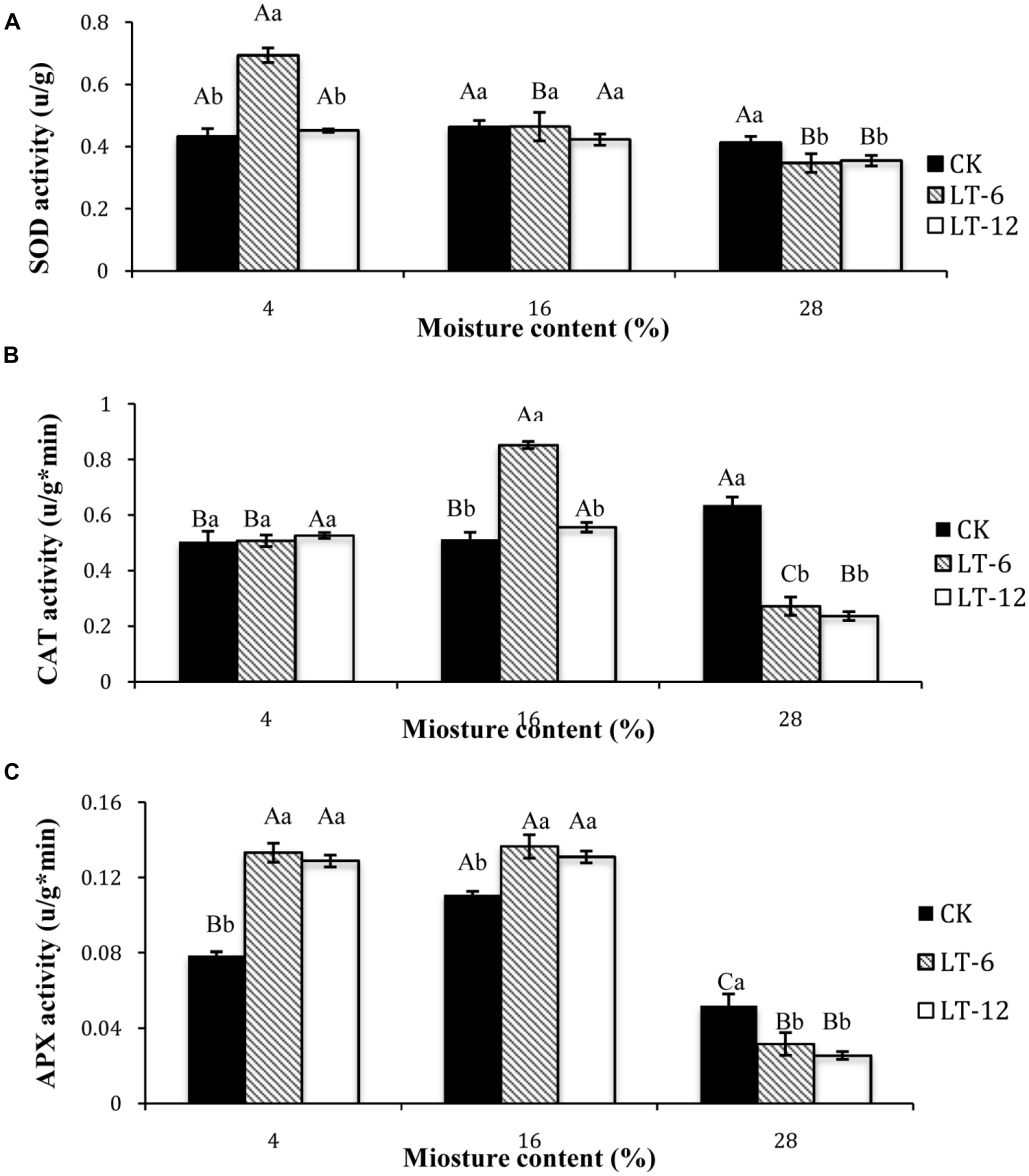
**Effect of storage duration on SOD, CAT, and APX activity of oat seed with different MC at 4°C. (A)** SOD; **(B)** CAT; **(C)** APX. The different letters indicate significant differences at 0.05 level among treatments as determined by the Duncan’s multiple range test. Means with different capital letters indicate the significant differences of seed at different MC and same storage duration, and with different lowercase letters at the same MC and different storage duration. Data represent the mean ± SD of four or more replicates.

In the storage treatments of CK, there was no significant difference (*P* > 0.05) for SOD activity of seeds among different MC (**Figure 3A**). In LT-6 and LT-12, the SOD activity significantly (*P* < 0.05) decreased with MC increasing from 4 to 28%. For seeds with 4% MC, SOD activities in CK and LT-12 were significantly (*P* < 0.05) lower than that in LT-6. For seeds of 16% MC, there were no significant (*P* < 0.05) differences for SOD activity among storage treatments. For seeds with 28% MC, SOD activity in LT-6 or LT-12 was significantly (*P* < 0.05) lower than that in CK.

In the storage treatments of CK, the CAT activity of seeds with 28% MC was significantly (*P* < 0.05) higher than that with 4 or 16%. In LT-6, the highest CAT activity occurred in seeds with 16% MC, and the lowest was in 28% MC. In LT-12, CAT activity in seeds of 28% MC decreased significantly (*P* < 0.05). For 4% moisture seeds, there were no significant (*P* > 0.05) differences for CAT activity among CK, LT-6, and LT-12. For 16% moisture seeds, the maximum CAT activity appeared after stored 6 months. For 28% moisture seeds, CAT activity in CK was significantly (*P* < 0.05) higher than other treatments (**Figure 3B**).

The changes of APX activity in oat seeds during storage showed that the highest APX activity in CK occurred in 16% moisture seeds, and the lowest was in those with 28% MC (**Figure 3C**). In LT-6 and LT-12, APX activities decreased significantly (*P* < 0.05) in 28% moisture seeds. For seeds with 4 and 16% MC, APX activities in LT-6 and LT-12 were significantly higher (*P* < 0.05) than that in CK. In seeds with 28% MC, changing of APX activity was opposite (**Figure 3C**).

### Changes in Transcript Levels of Genes Encoding Antioxidant Enzymes in Oat Seeds During Storage

**FIGURE 4:**
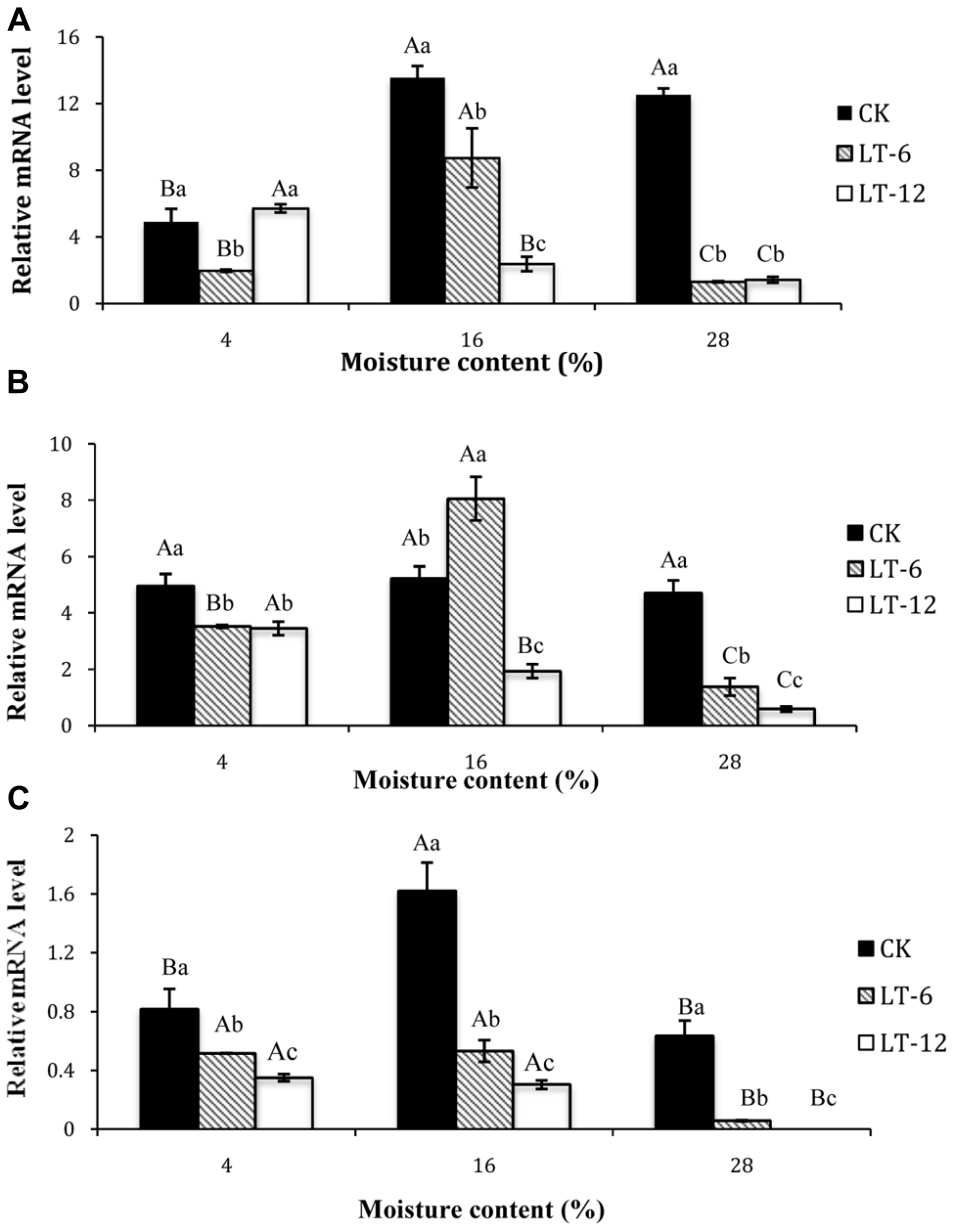
**Effect of storage duration on *SOD1, CAT1* and *APX1* gene expression of oat seeds with different MC at 4°C. (A)** *SOD1*; **(B)** *CAT1*; **(C)** *APX1.* The different letters indicate significant differences at 0.05 level among treatments as determined by the Duncan’s multiple range test. Means with different capital letters indicate the significant differences of seed at different MC and same storage duration, and with different lowercase letters at the same MC and different storage duration. Data represent the mean ± SD of three or more replicates.

According to the results of transcript levels in oat seeds with different MC (**Figure 4A**), the *SOD1* transcript level in CK was lowest in 4% moisture seeds. In LT-6, the highest *SOD1* transcript level presented in seeds of 16% MC. In LT-12, the *SOD1* transcript level were down-regulated significantly (*P* < 0.05) with MC increased from 4% to 28%. For seeds with 4% MC, *SOD1* in LT-6 was down-regulated and up-regulated in LT-12. In seeds with 16 and 28% MC, the levels of *SOD1* were down-regulated as the storage duration prolonged.

The *CAT1* transcript levels of seeds were similar among different MC in CK (**Figure 4B**). In LT-6 and LT-12, the changing of *CAT1* transcript level was similar with *SOD1*. The *CAT1* transcript levels tended to decrease with increasing storage duration in seeds with 4 and 28% MCs (**Figure 4B**). The transcript level of* CAT1* in seeds with 16% MC was up-regulated in LT-6, but down-regulated in LT-12.

The changes of *APX1* indicated that the highest *APX1* level in CK occurred in seeds of 16% MC, and the lowest was of 28% MC. The transcript levels of *APX1* in seeds with 4, 16, and 28% MC all decreased significantly (*P* < 0.05) during storage (**Figure 4C**).

### Changes in Proline Contents in Oat Seeds during Storage

**FIGURE 5:**
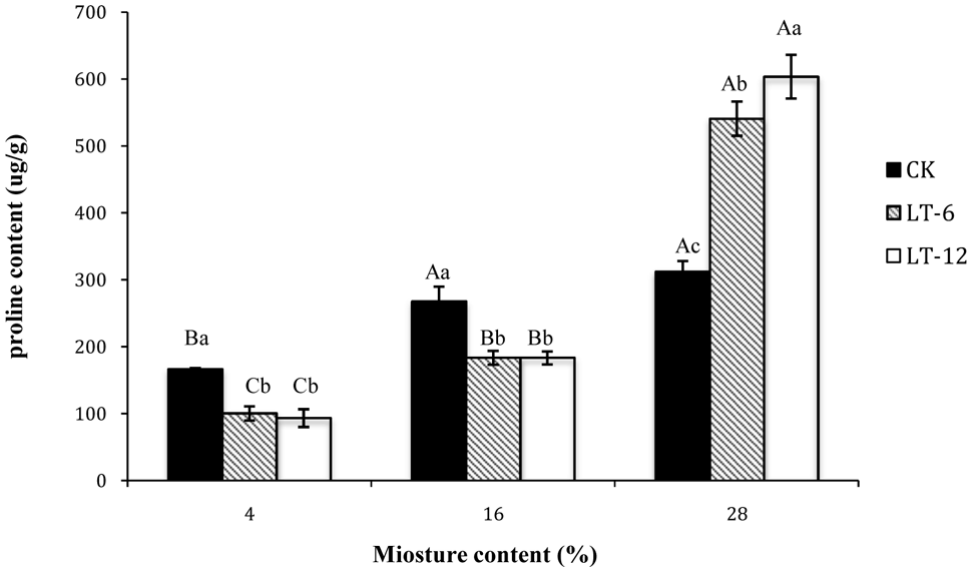
**Effect of storage duration on proline content of oat seed with different MC at 4°C**. The different letters indicate significant differences at 0.05 level among treatments as determined by the Duncan’s multiple range test. Means with different capital letters indicate the significant differences of seed at different MC and same storage duration, and with different lowercase letters at the same MC and different storage duration. Data represent the mean ± SD of four or more replicates.

The proline contents in LT-6 and LT-12 increased significantly (*P* < 0.05) with seed MC increasing from 4 to 28% (**Figure 5**). In CK, the proline contents of seeds with 4% MC was significantly (*P* < 0.05) lower than that with 16 and 28% MC. For seeds with 4 and 16% MC, there were no significant (*P* > 0.05) differences in proline content between LT-6 and LT-12, but both showed significantly (*P* < 0.05) lower proline contents than those in CK (**Figure 5**). For seeds with 28% MC, the proline content increased significantly (*P* < 0.05) with storage duration prolonged.

### Changes in Transcript Levels of Genes Related to Proline Biosynthesis and Catabolism during Storage

**FIGURE 6:**
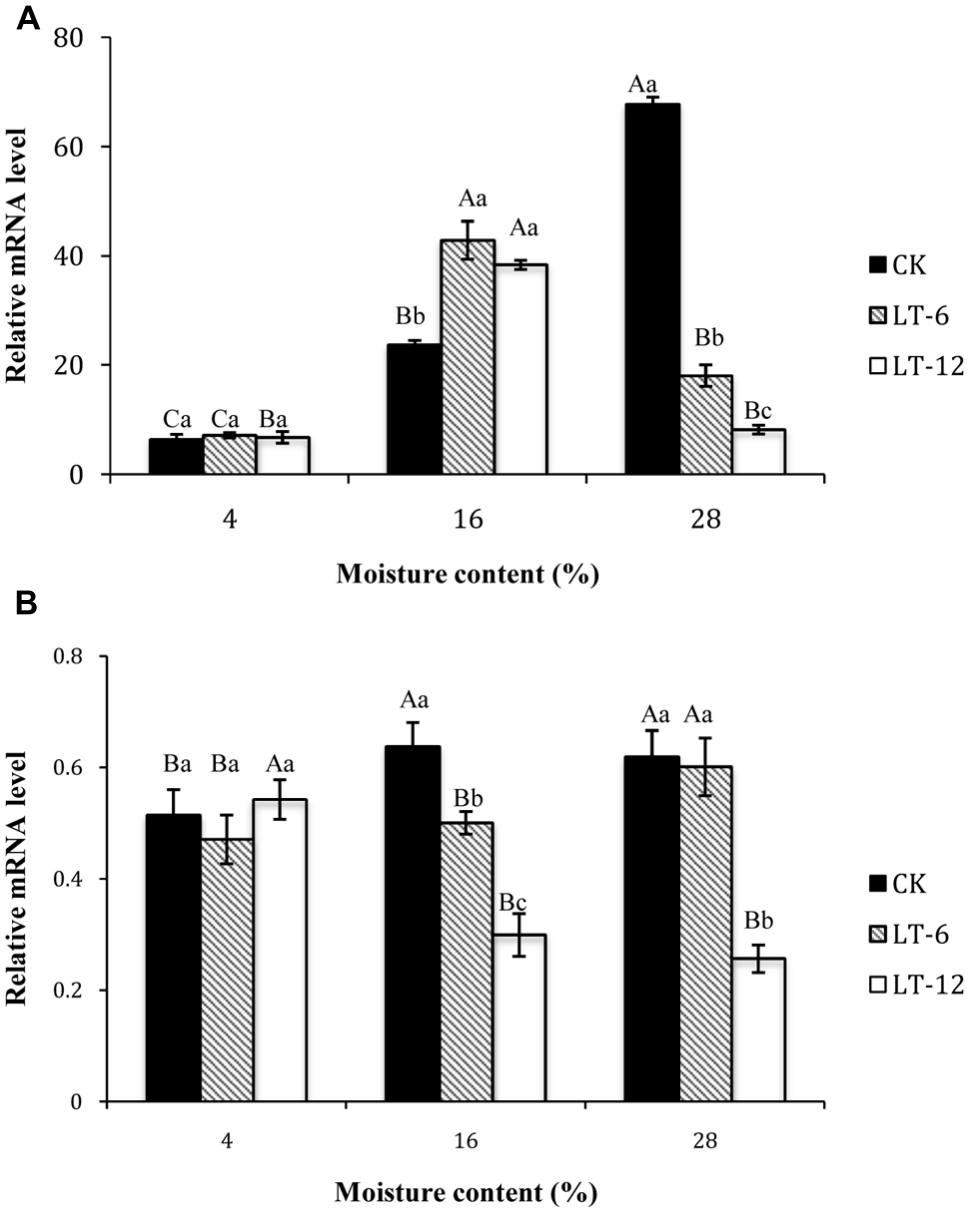
**Effect of storage duration on *P5CS1* and *PDH1* gene expression of oat seeds with different MC at 4°C. (A)** *P5CS1*; **(B)** *PDH1.* The different letters indicate significant differences at 0.05 level among treatments as determined by the Duncan’s multiple range test. Means with different capital letters indicate the significant differences of seed at different MC and same storage duration, and with different lowercase letters at the same MC and different storage duration. Data represent the mean ± SD of three or more replicates.

In the storage treatments of CK, the transcript level of *P5CS1* increased significantly (*P* < 0.05) with seed MC increasing from 4 to 28% (**Figure 6A**). In LT-6 and LT-12, the highest transcript level of *P5CS1* occurred in seeds of 16% MC. There was no significant (*P* > 0.05) differences for *P5CS1* transcript levels in seeds with 4% MC during storage. For seeds with 16% MC, the transcript levels of* P5CS1* in LT-6 and LT-12 were higher than that in CK. For seeds with 28% MC, the transcript levels of *P5CS1* reached the minimum level after stored 12 months.

The changing of transcript levels of *PDH1* during seed storage indicated that *PDH1* levels in CK and LT-6 both increased with MC increasing from 4 to 28% (**Figure 6B**), but there was opposite changing for the transcript levels of* PDH1* in LT-12. For seeds with 4% MC, there were no significant (*P* > 0.05) differences for transcript levels of* PDH1* among three storage treatments. For seeds with 28% MC, the transcript levels of *PDH1* decreased with storage duration prolonged, and reached the minimum after stored 12 months.

## Discussion

Seed MC and storage duration greatly affect seed longevity during storage (Abba and Lovato, 1999; Mira et al., 2015). In this study, the germination percentage of oat seeds with 28% MC decreased significantly during storage (**Figure 1**). As the storing from 0 to 12 months, there was no significant difference for germination of oat seeds with 4% MC, but seeds with 28% MC did not germinate well, even in the CK. These findings suggested that low MC might be more important than the storage duration in terms maintaining the vigor of stored seeds. Similar results have been reported for seeds of many species under hermetic storage (Ellis et al., 1988; Zhang et al., 2010). The optimum MC for oat seeds may be 4%, but 16% MC was acceptable for seeds stored for less than 6 months.

The loss of seed germinability has been attributed to the accumulation of ROS (Wojtyla et al., 2006). Many reports have indicated that oxidative stress imposed by ROS is an important cause of seed deterioration during aging (McDonald, 1999; Rajjou and Debeaujon, 2008). In our study, the reduction of seed vigor was related to higher levels of ROS, such as $O_{2}^{\cdot -}$ and H_2_O_2_, as seed MC increased from 4 to 28% (**Figure 2**). A loss of seed viability was shown to be positively correlated with increasing MC in seeds of sunflower, beech (*Fagus sylvatica*), holm oak (*Quercus ilex* L.) and cotton (*Gossypium hirsutum* L.; Bailly et al., 1996; Pukacka and Ratajczak, 2005; Kibinza et al., 2006). Higher MCs in aging seeds resulted in greater oxidative damage and ROS generation. In this study, as the storage duration extended from 0 to 12 months, the $O_{2}^{\cdot -}$ production rate remained stable in seeds with 28% MC. The lowest $O_{2}^{\cdot -}$ production rate in seeds with 4 and 16% MC was attained after 6 months of storage (**Figure 2A**). The H_2_O_2_ content showed a similar trend; that is, the minimum level was in seeds with 4% MC after 6 months of storage (**Figure 2B**). These differences in H_2_O_2_ contents might be related with the level of MC. However, antioxidant enzymes could be activated after 6 months storage or the contents of non-enzymatic antioxidants had increased in seeds with 4 and 16% MC. This result suggested that seeds with lower MCs were able to repair the deteriorated damages at 4°C and storage of 6 months.

Antioxidant enzymes and non-enzymatic antioxidants, including SOD, CAT, APX, and proline, contribute to reduce the concentration of ROS (Kishor et al., 1995; Mittal et al., 2012; Oliveira et al., 2012). SOD is one of the most important enzymes in cellular defense against ROS, as it could catalyze the $O_{2}^{\cdot -}$ into H_2_O_2_ (Kumar et al., 2010; Kumara et al., 2010). CAT and APX have been demonstrated to scavenge the H_2_O_2_ produced by interacting under oxidative stress (Erdal et al., 2011; Yin et al., 2014). CAT reduces H_2_O_2_ to water and dioxygen, and APX reduces H_2_O_2_ to water and generates MDA (Noctor and Foyer, 1998). Activities of antioxidant enzymes have been observed to decrease in aged soybean, cotton, and sunflower (Bailly et al., 1996; Murthy et al., 2002; Goel et al., 2003). Increasing of MC promotes enzymatic oxidation and leads to rapid cellular damage, which decreases seed vigor (Gill and Tuteja, 2010). In this study, the changing of SOD activity was related with the level of MC and storage duration. There were no significant changes in CK with MC increasing, however, SOD activity decreased with MC increasing as stored 6 and 12 months (**Figure 3A**). There was different changing tendency between CAT and APX activity with MC increasing and storage duration prolonged. CAT and APX could respond rapidly to scavenge H_2_O_2_ under stress. CAT activity was more responsive to the variation in higher MC compared to APX. The highest SOD activity in seeds with 4% MC occurred after 6 months of storage, but the $O_{2}^{\cdot -}$ production rate and H_2_O_2_ content reached the lowest level. Furthermore, the different changing for CAT and APX activity under moisture of 4% indicated that APX was more responsive than CAT to oxidative damage as the storage duration extended. These two enzymes may have complementary or interacting roles. The activities of SOD, CAT, and APX in seeds with 28% MC showed similar trends. The reductions in SOD, CAT, and APX activities may be because of ROS accumulated to toxic levels (Kong et al., 2014) or intolerant of the higher MC and/or long-term storage.

The accumulation of low-molecular weight metabolites acting as osmoprotectants, such as proline, is part of the adaptive response to stress in plants (Hoekstra et al., 2001). Kishor and Dange (1990) reported that proline protected cellular functions by scavenging ROS. In our study, proline content tended to increase with seed MC increasing. It’s illustrated that proline accumulation in seeds could confer some adaptive advantages under stress. However, an increasing in proline content could also be considered as a stress-induced marker for oxidative damage during aging (Lei and Chang, 2012). As seeds storing 12 months, the proline contents decreased in seeds with 4 and 16% MC, but increased in seeds with 28% MC (**Figure 5**). Meanwhile, antioxidant enzyme activity also tended to increase in seeds with 4 and 16% MC, but decrease in those with 28% content MC during storage (**Figure 3**). This result suggested that proline played a more important role in adapting to oxidative stress in seeds with higher MC (28%), while antioxidant enzymes played a more important role in seeds with lower MCs (4 and 16%).

To elucidate the role of the ROS scavenging system during seed aging, we analyzed the transcript levels of genes encoding antioxidant enzymes by real-time PCR. The changes in transcript levels of *SOD1* which encodes a Cu/Zn SOD were not consistent with the SOD activity (**Figure 4A**). It indicated that SOD1 was not the main enzyme contributing to total SOD activity. Vaseva et al. (2012) proposed that Cu/Zn SOD was suppressed by Fe SOD in aging oat seeds. The transcript levels of* APX1* and *CAT1* in seeds with different MC were consistent with APX and CAT activities during storage, suggesting that APX1 and CAT1 were the major H_2_O_2_-scavenging enzymes for maintaining the balance of redox reaction in aged oat seeds. From these results, the expressions of APX1 and CAT1 in oat seeds were suppressed during aging and leading to H_2_O_2_ accumulation. Similar results have been reported for rice seeds (Yin et al., 2014).

*P5CS1* catalyzes the first step of proline synthesis (Abraham et al., 2003). Overexpression of *P5CS1* improved stress tolerance in rice and wheatgrass (Choudhary et al., 2005). In this study, *P5CS1* transcript levels were significantly up-regulated with MC increasing from 4 to 28% in CK (**Figure 6A**). However, the expression of *P5CS1* presented the up-regulation at 16% MC and down-regulation at 28% during 6 and 12 months storage, while the proline content increased continually. This indicated that *P5CS1* transcript levels were affected significantly by the seed MC. Some studies have shown that suppression of *PDH1* increased the proline content and enhanced stress resistance (Nanjo et al., 1999; Armengaud et al., 2004). In our study, *PDH1* transcript levels showed the opposite trend in comparison with proline contents in oat seeds stored 12 months, and reached lower levels at MCs of 16 and 28%. This implied that PDH1 could improve stress resistance for seed aging and maintain seed vigor during long-term storage.

In summary, proline played the main role in adaptation to oxidative stress in seeds with higher MC (28%), while antioxidant enzymes (SOD, CAT, APX) played the main roles in seeds with lower MCs (4%, 16%) during storage at a low temperature (4°C). The transcript analyses showed that *SOD1* was not a main factor in total SOD activity, while *APX1* and *CAT1* were the main H_2_O_2_-scavenging enzymes in aging oat seeds. The transcript level of *P5CS1* was significantly affected by MC, and *PDH1* could improve stress resistance for seed aging and maintain seed vigor during long-term storage.
